# Correlation between plasma ccfDNA, mtDNA changes, CTCs, and epithelial-mesenchymal transition in breast cancer patients undergoing NACT

**DOI:** 10.55730/1300-0144.5834

**Published:** 2024-03-11

**Authors:** Betül ÇELİK, İrem PEKER EYÜBOĞLU, Sinan KOCA, M. Ümit UĞURLU, Özkan ALAN, Gökçe GÜLLÜ AMURAN, Tuğba AKIN TELLİ, Fulden YUMUK, Mustafa AKKİPRİK

**Affiliations:** 1 Department of Medical Biology and Genetics, Health Sciences Institute, Marmara University, İstanbul, Turkiye; 2 Department of Medical Biology, School of Medicine, Marmara University, İstanbul, Turkiye; 3 Department of Medical Oncology, Ümraniye Education Research Hospital, İstanbul, Turkiye; 4 Department of General Surgery, School of Medicine, Marmara University, İstanbul, Turkiye; 5 Department of Medical Oncology, School of Medicine, Marmara University, İstanbul, Turkiye

**Keywords:** Breast cancer, ccfDNA, mtDNA, neoadjuvant therapy, EMT

## Abstract

**Background/aim:**

Breast cancer is the most prevalent cancer in women, emphasizing need for noninvasive blood biomarkers to aid in treatment selection. Previous studies have demonstrated elevated levels of plasma circulating cell-free DNA (ccfDNA) in breast cancer patients. Both ccfDNA and mitochondrial DNA (mtDNA) are fragments released into the bloodstream. In this study, we investigated effectiveness of ccfDNA and mtDNA as indicators of treatment response and explored their potential as monitoring biomarkers. Additionally, we compared these markers with circulating tumor cell (CTC) data and assessed their relationship with epithelial-mesenchymal transition (EMT).

**Materials and methods:**

Thirty-six female breast cancer patients and 21 healthy females were included in the study. Quantitative polymerase chain reaction (qPCR) was performed on plasma samples to measure levels of ND1, ND4, ALU115, ALU247, and GAPDH, and DNA integrity was determined by calculating ratios of ALU247/ALU115 and ND4/ND1.

**Results:**

After treatment, patients had a significant decrease in ccfDNA levels and a significant increase in mtDNA copy number (mtDNAcn). However, there was no significant change in ccfDNA and mtDNA integrity. When comparing all groups, patients exhibited higher levels of ALU115 and ALU247 compared to controls. Moreover, patients demonstrated significantly lower ccfDNA integrity than controls.

**Conclusion:**

This study represents the first comprehensive investigation of plasma ccfDNA levels, mtDNAcn, and integrities collectively. Furthermore, it is the first study to explore the relationship between these markers and CTCs, cancer stem cell markers, treatment response, and metastatic status. Our findings suggest that plasma ccfDNA and mtDNA may serve as potential biomarkers for assessing chemotherapy response and can be employed alone or in combination with other biomarkers to monitor treatment efficacy in breast cancer patients.

## 1. Introduction

Breast cancer is the most frequent cancer worldwide and ranks as the second leading cause of cancer-related death in women [1]. Multiple molecular changes that lead to uncontrolled self-renewal, proliferation, transformation, and metastasis of normal cells cause cancer [2,3]. Early detection of cancer and accurate identification of metastases have considerably improved the survival rates of women with breast cancer by enhancing treatment options. Monitoring treatment response is crucial to prevent the continuation of ineffective treatments, minimize unnecessary side effects, and assess the effectiveness of new therapeutics [1,2,4,5]. Accumulating findings over the last couple of years have emphasized the potential use of circulating nucleic acids in peripheral blood, such as DNA, mRNA, and microRNA, in the breast cancer diagnosis, prognosis, and monitoring of response to anticancer therapy [6]. While the use of tumor tissue specimens remains important, the utility of biopsy samples is limited because they may not capture tumor heterogeneity, and recurrent biopsies are not practical. A considerable alternative method is “liquid biopsy”, which allows for sensitive and targeted serial sampling during therapy [5].

Changes in the levels of circulating tumor cells (CTCs), extracellular free DNAs (cfDNAs), and mitochondrial DNA (mtDNA), known as liquid biopsies, have received great attention as cancer biomarkers in plasma and serum. In addition to plasma and serum, liquid biopsies are a minimally invasive tool for detecting molecular biomarkers in body fluids such as peripheral blood, urine, saliva, cerebral spinal fluid, and breast milk [7–13]. This approach provides a perspective for real-time monitoring of tumor dynamics in an individual cancer patient. While tissue biopsy from a cancerous location remains the mainstay of diagnosis, liquid biopsy presents an alternative to the restricted solid biopsy approaches due to several advantages, including the ability to perform sequential sampling for monitoring tumor progression, treatment response, metastasis, and disease recurrence [4,9,11].

In 1948, two French researchers, Mandel and Métais, discovered the presence of cell-free DNA in the blood of both healthy and diseased humans [7,10,11,14–19]. While cfDNA might have seemed insignificant when it was first discovered in the human circulatory system, its clinical significance was recognized when researchers observed differences between the properties of cfDNA in healthy individuals and cancer patients [16]. The vast majority of cfDNA is released through apoptosis or necrosis of tumor cells in oncological patients [20,21]. Besides screening healthy and at-risk patient groups for early detection and cancer treatment, cfDNA serves as a biomarker for multiple indications in oncology, including staging and prognosis, tumor localization, initial therapy stratification, monitoring of local or systemic treatment response, identification of acquired resistance mechanisms, monitoring of recurrence [8,16,22].

The cfDNA profile found in a single blood sample contains a mixture of both “wild-type” and genetically and epigenetically modified DNA fragments released by diverse cells from various processes, tissues, and organs under environmental factors [23]. All cells seem to have the ability to continuously release cell-specific DNA into the extracellular environment. An important point here is that the concentration of cfDNA and the concentration of tumor-derived DNA in tumor microenvironment, as well as in other healthy cells, can vary significantly between individuals [16].

cfDNA presents as ALU (*Arthrobacter luteus*) sequences. More than 50%–65% of the human genome consists of repetitive DNA [18,24]. ALU families belonging to the class of retroelements called short interspersed nuclear elements (SINEs) in the more than 10% of the human genome, with a copy number of approximately 1.4 million, are the most abundant ALU [18,25]. They are typically about 300 nucleotides in length [14,26–28]. While the source of cfDNA in healthy individuals is merely by apoptosis, producing evenly sized shorter DNA fragments (ALU 115); on the other hand, in cancers, necrosis contributes uneven longer DNA fragments (ALU 247) to the shorter fragments from apoptosis [14,29–31]. Analysis of ccfDNA integrity is a factor independent of the genetic or epigenetic status of cfDNA and is theoretically representative of all tumors. ccfDNA integrity is calculated as the ratio of the concentration of longer DNA fragments to shorter fragments in plasma or serum [32,33].

Mitochondria are eukaryotic cell organelles that play a central role in energy production, cell proliferation, and apoptosis. It is the main source and target of intracellular reactive oxygen species (ROS), which plays an important role in breast carcinogenesis [34]. Recent advances aimed at increasing the diagnostic and prognostic value for cancer patients have also targeted the circulating mitochondrial genome due to its unique and distinctive properties. Circulating mitochondrial DNA is known to have short length, relatively simple molecular structure, and high copy number. These properties make it an easily accessible, noninvasive biomarker for diagnosing various types of solid tumors, complementing the function of liquid biopsy [35]. In addition to the inconsistent association between peripheral blood mtDNA copy number and breast cancer risk, breast cancer may alter the observed mtDNA levels in peripheral blood, emphasizing the need for designing forward-looking studies [36].

There are hotspot locations for deletions along the mtDNA circle, 90% of which are deletions of the nicotinamide adenine dinucleotide dehydrogenase 4 (ND4) sequence, reflecting a population of viable mitochondria but with poor mtDNA integrity [37]. ND4 subunits are often missing in complex I and are a common indicator of mtDNA damage [38]. In contrast, the loss of ND1 subunits has a much more detrimental effect on complex I and the mitochondria itself, making ND1 deletions rare in viable mitochondria. Therefore, the rarely deleted ND1 copy number is a suitable marker for the total mtDNA copy number and the ND4/ND1 ratio can be used to assess the proportion of intact mtDNA [37–40].

Breast cancer treatment typically incorporates a multimodality strategy that includes surgery, radiation, and systemic therapy. Neoadjuvant chemotherapy (NACT) has become a standard treatment for patients with advanced breast cancer. If breast-conserving surgery is not possible, neoadjuvant chemotherapy can be utilized [41]. It has become a standard-of-care for patients with locally advanced breast cancer. NACT provides a unique opportunity for real-time monitoring of tumor response and evaluation of drug efficacy. Secondly, it can reduce the stage of tumors and thus promote the chances of breast-conserving surgery [42–44].

Currently, the need for diagnostic and prognostic biomarkers continues. However, using a combination of blood biomarkers with a noninvasive method is critical for treatment selection. According to the studies reviewed, ccfDNA and mtDNA play crucial roles in human cancers, particularly in their significant involvement in metastasis. Given the lack of research on the relationship between multiple markers, circulating tumor cells, and epithelial-mesenchymal transition, the present study aims to contribute to clinical research in disease diagnosis, prognosis, and treatment.

In this study, we investigated the changes in plasma cfDNA and mtDNA copy number and integrity before and after NACT in breast cancer patients. By correlating these findings with CTC molecular analysis data obtained from the same patients in our previous study, we aimed to examine the relationship of these biomarkers with epithelial-mesenchymal transformation (EMT). This approach aims to predict the likelihood of metastasis development and consequently forecast the clinical course of these patients.

## 2. Materials and methods

### 2.1. Study subjects and ethical approval

Breast cancer patients receiving neoadjuvant chemotherapy (n = 36) and healthy individuals as control (n = 21) were included in this retrospective study. Plasma retrieved from specimens stored at −80 °C. The present study was approved by the Marmara University School of Medicine Ethics Committee (approval ID 09.2022.246). Informed consent was obtained from all the recruited subjects.

### 2.2. Isolation of DNA

Blood samples were collected in EDTA tubes. To obtain plasma, centrifugation was performed at 2000 × *g* for 10 min at 4 °C. DNA was extracted from plasma samples using the QIAmp DNA mini kit (Qiagen, Hilden, Germany, cat. number: 51304) according to the manufacturer’s instructions and stored at −20 °C until use.

### 2.3. RT-PCR of ALU elements and mtDNA copy number

One microliter of genomic DNA was used as a template for each real-time polymerase chain reaction (RT PCR) using BlasTaq 2X SYBR Green Master Mix (abm Good) on a real-time PCR device (LightCycler 480, Roche).

The quantitative values obtained from the 115 bp (shorter fragments) primers represent the total level of ccfDNA (ng/μL), while the quantitative values from the 247 bp (longer fragments) primers were used to calculate the integrity of ccfDNA. The sequences of the ALU 115 and ALU 247 primers were as follows: ALU 115 forward 5′-CCTGAGGTCAGGAGTTCGAG-3′ and reverse 5′-CCCGAGTAGCTGGGATTACA-3′; ALU 247 forward 5′-GTGGCTCACGCCTGTAATC-3′ and reverse 5′-CAGGCTGGAGTGCAGTGG-3′ [45]. The absolute amount of ccfDNA in each sample was determined by a standard curve using 10-fold dilutions (10, 1, 0.1, 0.01, 0.001) of genomic DNA obtained from peripheral blood of a healthy donor volunteer. A negative control (without template) was run in each reaction plate.

We analyzed the levels of ND1, ND4, and GAPDH to determine mtDNA copy numbers in plasma samples. ND1 copy number serves as a convenient marker for the total amount of mtDNA. The sequences of the ND1 and ND4 were obtained from NCBI. The ND1 sequences were as follows: forward 5′-ATGGCCAACCTCCTACTCCT-3′ and reverse 5′-GGGCCTTTGCGTAGTTGTAT-3′. The ND4 sequences are: forward 5′-GATGAGGCAACCAGCCAGAA-3′ and reverse 5′-GTAGGGGAAGGGAGCCTACT-3′. After obtaining the Ct values, the mtDNA copy number was calculated using the following formula: 2^−DCt^.

Real-time PCR amplification was performed with the following cycles: Initial holding at 95 °C for 3 min, followed by 50 cycles of 95 °C for 15 s and 60 °C for 60 s. Negative template control was run in each plate. All reactions were conducted in triplicate in 96-well plates.

### 2.4. DNA integrity determination

DNA integrity was calculated as the ratio of ALU 247 to ALU 115 for ccfDNA and the ratio of ND4 to ND1 for ccf-mtDNA. In this study, mtDNA integrity was calculated by proportioning the amounts obtained as a result of the 2^−DCt^ formula of the ND1 and ND4 primer sets while the ratio of longer to shorter fragments (ALU 247 (ng/μL) / ALU 115 (ng/μL)) demonstrates the integrity of ccfDNA in each sample [14,38,45].

### 2.5. Statistical analysis

In this study, analysis was conducted on patients who received neoadjuvant chemotherapy before treatment, after treatment, and a control group. The SPSS 17.0 program was used to examine levels of ccfDNA, ccfDNA integrity, mtDNA copy number, and mtDNA integrity. The results were assessed individually using the Wilcoxon signed-rank test and collectively using the Mann–Whitney U test. A significance level of p < 0.05 was considered unless otherwise specified for statistical analyses.

## 3. Results

In this study, data were obtained from 34 women before treatment and 30 women after treatment with locally advanced breast cancer. As a control group, 21 age-matched healthy volunteers were included in the study. The mean ages of the participants were 50.35 (32–80) years. The age range of the control group was less than 50 years. The clinicopathologic characteristics of the patients before and after NACT were given in our previous study and Table 1 [41].

### 3.1. ccfDNA amount and integrity index at pre- and posttreatment

**Figure 1:**
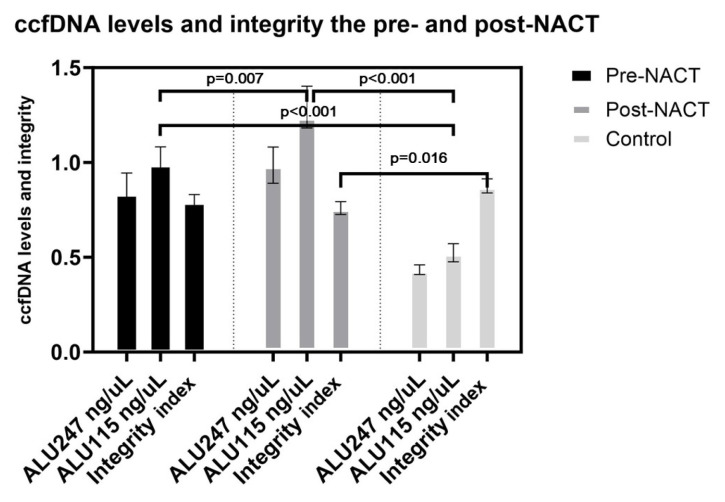
ccfDNA levels and integrity the pre- and post-NACT.

While the mean ALU values increased, the ccfDNA integrity index decreased posttreatment. The ALU values of the healthy controls were lower than the pre- and posttreatment values (p < 0.01) (Figure 1).

### 3.2. mtDNA amount and integrity index at pre- and posttreatment

**Figure 2:**
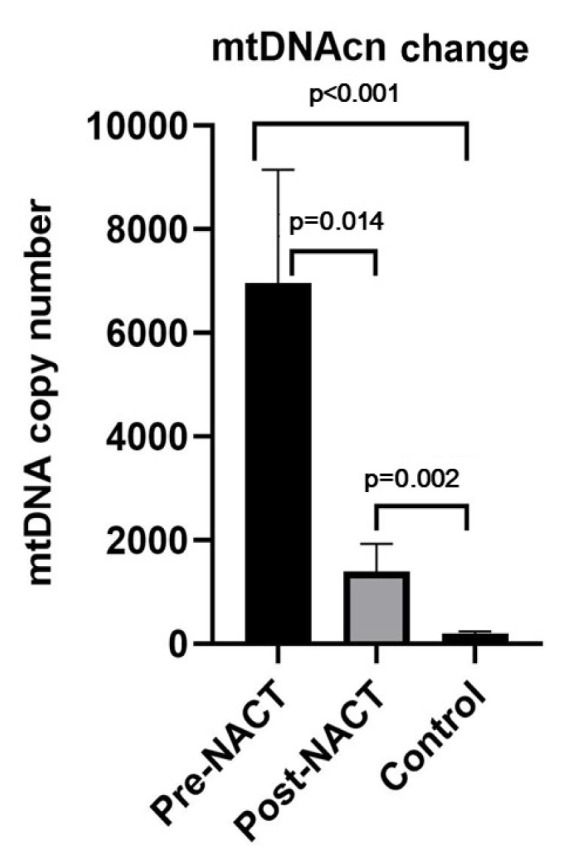
mtDNAcn the pre- and post-NACT.

**Figure 3:**
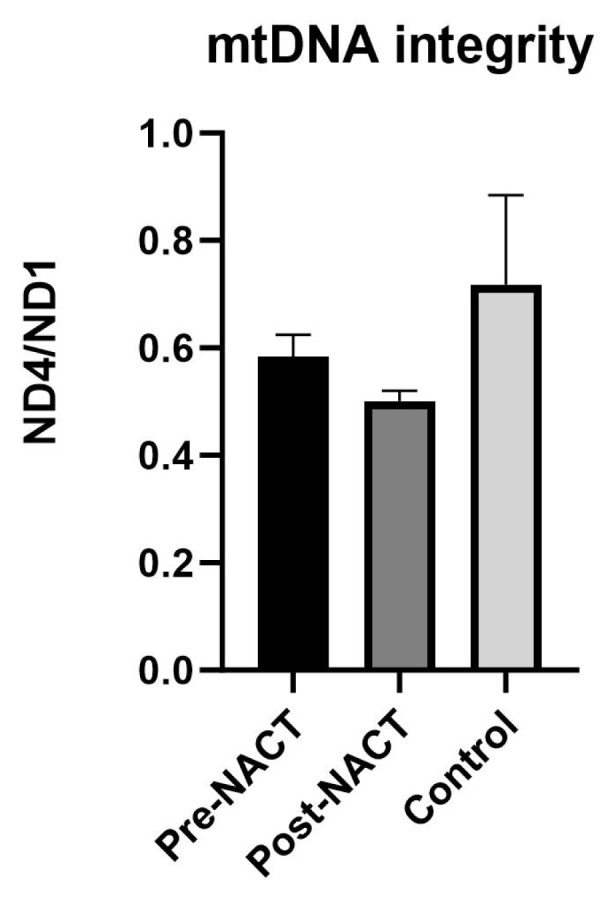
mtDNA integrity the pre- and post-NACT

Analysis of the pretreatment and post-treatment groups revealed a significant reduction in mtDNA copy number in patients prior to treatment compared to posttreatment group (mean: 6956.58 to 1395.16, p = 0.014) (Figure 2). Following treatment, an average decrease in mtDNA integrity from 0.58 to 0.50 was observed, although statistical significance was not reached (p = 0.135) (Figure 3).

The mtDNA copy number pretreatment was found to be higher than in the control group, which was statistically significant (p < 0.001) (Figure 2). Analysis of the mtDNA integrity index revealed a value of 0.58 in breast cancer patient’s pretreatment. In contrast, the control group demonstrated a mean integrity index of 0.72. However, statistical analysis showed no significant difference between the two groups (p = 0.690) (Figure 3).

When we examine the difference between the posttreatment and control groups; the mtDNA copy number posttreatment was approximately 7.34 times higher than the that in the control group (p = 0.002) (Figure 2). However, the mean mtDNA integrity index was 0.72 in the control group, while it was 0.50 in the posttreatment group, but this difference was not statistically significant (p = 0.528) (Figure 3).

### 3.3. The results of Wilcoxon signed-rank test at pre- and posttreatment

**Figure 4:**
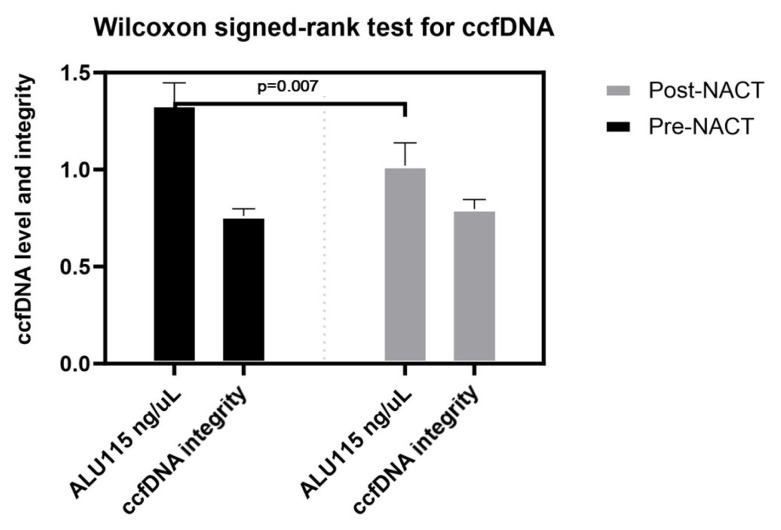
Wilcoxon signed-rank test for ccfDNA.

We have pre- and posttreatment data for only 28 of 34 patients. When examine the pre- and posttreatment results of this patient group, the posttreatment ccfDNA levels were lower than those of the pretreatment group, and the difference was statistically significant (p = 0.007). The ccfDNA integrity index of the posttreatment group was higher than that of the pretreatment group, but the difference was not statistically significant (p = 0.665) (Figure 4).

**Figure 5:**
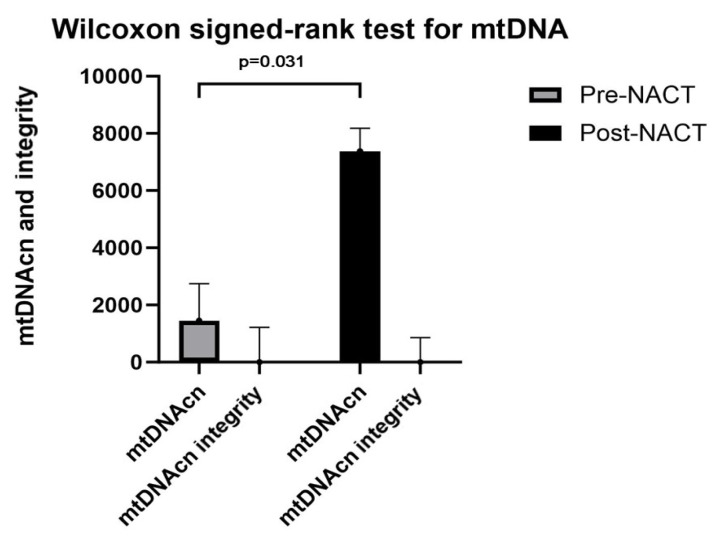
Wilcoxon signed-rank test for mtDNA.

The mtDNA copy number was significantly higher in the posttreatment group than in the pretreatment group (p = 0.031). The mtDNA integrity index in the posttreatment group was higher than that in the pretreatment group, while this difference was not statistically significant (p = 0.820) (Figure 5).

### 3.4. ccfDNA, mtDNA, CTC, EMT, and ALDH1 at pre- and posttreatment

We aimed to examine the relationship between changes in plasma cfDNA and mtDNA copy numbers and integrity with CTC, EMT, and ALDH1 data obtained in a previous study by our team [41]. Detailed results of CTC, EMT, and ALDH1 pre- and post- NACT were presented in our previous study [41]. All cfDNA and mtDNA results, along with other biomarker findings, were given in Table 2. There was no correlation found between ccfDNA levels and mtDNA copy number with CTC, EMT, and stem cell markers pre- and post-NACT. The results were not statistically significant (p > 0.05).

### 3.5. Relationship of ccfDNA levels and mtDNAcn with breast cancer type, therapy response, and metastasis

When we compare the same patient group with previous study data, only 1 out of 6 patients who tested negative for metastasis and exhibited complete pathological and clinical responses showed negative CTC, EMT, and ALDH1 markers both before and after NACT. However, mtDNA copy number decreased, while ccfDNA level increased after treatment, but the difference was not statistically significant (p > 0.05). Patients’ ccfDNA and mtDNA levels were analyzed according to breast cancer type. No significant results were found between them (p > 0.05) (Table 2).

## 4. Discussion

Breast cancer stands as the leading form of cancer affecting women globally, presenting numerous challenges for effective treatment [1]. Traditional methods such as tissue biopsy, although widely employed, have limitations in terms of comprehensively detecting the disease and monitoring treatment response. Additionally, the site of metastases can pose obstacles to biopsy procedures [9,16]. Therefore, there is a critical clinical need for noninvasive biomarkers that can aid in the diagnosis and follow-up of breast cancer [46].

Breast cancer exhibits a complex molecular landscape, necessitating innovative approaches for its detection and monitoring. The advent of liquid biopsy, which involves the analysis of circulating tumor cells (CTCs), cell-free DNAs (cfDNAs), and mitochondrial DNA (mtDNA) in plasma and serum, has emerged as a promising noninvasive tool in cancer diagnostics [9,16,47]. Cancer cells can enter the bloodstream early in the disease process, even before the detection of a tumor, and can disseminate throughout the body. By capturing and analyzing CTCs, cfDNAs, and mtDNA released through apoptosis, necrosis, or active release during tumor growth, liquid biopsy enables the comprehensive assessment of disease dynamics [47].

Plasma ccfDNA and mtDNA were evaluated as blood biomarkers to assess neoadjuvant chemotherapy response in breast cancer patients. Changes in ccfDNA levels, mtDNA copy numbers, and integrity were detected. The relationship between CTC molecular analysis and EMT was explored, aiming to predict metastasis development and estimate patient outcomes.

Consistent with previous findings, breast cancer patients exhibited higher plasma levels of circulating cell-free DNA (ccfDNA) compared to controls, alongside elevated levels of ALU 115 and ALU 247. This is likely due to increased release of fragmented DNA from apoptotic and necrotic cells in breast cancer patients. Similarly, a study involving breast and prostate cancer patients reported a higher ccfDNA integrity index in prostate cancer patients compared to controls, while breast cancer patients had a lower index. The amount of cfDNA released into circulation is influenced by factors such as cancer stage, tumor mutation load, and DNA clearance rate. The ALU DNA integrity index has been suggested as a more advantageous marker than absolute ccfDNA levels, as it correlates with tumor cell death. However, variations in DNA integrity index among different cancer types and individual cases highlight its heterogeneity and complexity. When we examined all the patients we have, ccfDNA levels increased significantly posttreatment, while the ccfDNA integrity index decreased and was not statistically significant. Severe destruction of cells and different number of pre- and posttreatment data may affect this situation. In studies conducted with various cancer patients differences in the integrity index were observed while ccfDNA levels increased posttreatment [43,45,48,49]. These findings highlight the importance of ALU 115 and ALU 247 levels, as well as ccfDNA integrity, as potential biomarkers for breast cancer.

In 28 patients with both pre- and posttreatment data, a significant decrease in ccfDNA levels and a nonsignificant increase in ccfDNA integrity were observed posttreatment. These findings align with a study by Adusei et al., who also reported a decline in serum ALU 247 and ALU 115 levels and an increase in ccfDNA integrity after three cycles of chemotherapy in breast cancer patients [14]. These findings emphasize the dynamic nature of plasma ccfDNA levels in response to treatment in breast cancer patients. The significant decrease in ccfDNA levels following treatment indicates its potential as a monitoring tool for treatment response. Further investigations are warranted to explore the underlying mechanisms driving the alterations in ccfDNA levels and integrity, and to evaluate their clinical implications in breast cancer management.

Chemotherapy treatment leads to the destruction of cancer cells, resulting in the release of cellular DNA into the bloodstream and subsequently increased levels of circulating cell-free DNA (ccfDNA) in the blood [48]. However, posttreatment measurements reveal lower ccfDNA levels, indicating a reduction in cancer cell population and activity, as well as an efficient clearance system for cfDNA. The extensive destruction of cancer cells during treatment may contribute to the release of longer DNA fragments, potentially affecting DNA integrity [14]. Studies in breast and colorectal cancer patients have demonstrated a decrease in ALU 115 levels and an increase in integrity following treatment [31,50,51]. These observations highlight the dynamic nature of ccfDNA and its potential as a valuable biomarker in monitoring chemotherapy response.

Our findings revealed a higher mitochondrial DNA (mtDNA) copy number in breast cancer patients compared to controls, with the highest levels observed pretreatment. These results were statistically significant. In contrast, mtDNA integrity was higher in the control group, although this difference was not statistically significant. Studies have reported conflicting results regarding mtDNA copy number, with some showing higher levels in the patient group and others demonstrating lower levels [52–58]. Consistent with our results, a study in breast cancer patients found the highest mtDNA copy number in late-stage cancer patients, while healthy individuals and early-stage cancer patients exhibited lower levels [59]. Additionally, elevated ND1 levels have been observed in thyroid and colorectal cancer patients compared to normal individuals, potentially indicating increased replication-induced mtDNA damage and the need for compensatory mtDNA molecules in tumor tissues [38,40,57]. Despite the mtDNA integrity index not reaching statistical significance and being lower in patients due to fragmented DNA and increased copy number, further research could explore its potential as a marker for treatment follow-up in breast cancer.

In our current study, we also aimed to examine the relevance of the previous findings to our research. However, we did not observe any statistically significant associations between ccfDNA levels, mtDNA copy numbers (mtDNAcn), and CTCs, EMT, ALDH1, treatment response, or metastasis. To gain a comprehensive understanding of these biomarkers’ clinical significance, further investigations involving larger patient cohorts are warranted. Serial monitoring and characterization of these biomarkers at specific time points during treatment are essential to elucidate their potential as clinically meaningful indicators. This study is the first to comprehensively investigate plasma ccfDNA levels, mtDNA copy number, and their integrities simultaneously. It is also the first to explore the relationship between these biomarkers and CTCs, cancer stem cell markers, treatment response, and metastatic status. Differences in biomarker levels observed in our study may stem from variations in factors such as blood collection periods, sample preprocessing, storage, DNA isolation procedures, as well as clinical characteristics including tumor stage, tumor size, and patient age. The findings highlight the potential of ccfDNA and mtDNA as biomarkers for monitoring chemotherapy response in breast cancer. Noninvasive methods for cancer detection and monitoring have gained significant attention in recent years. These approaches offer the potential for improved patient comfort, reduced invasiveness, and enhanced accessibility. The development of reliable biomarkers that can be detected through noninvasive means is crucial to address these clinical needs. However, due to the limited dataset and lack of pre- and posttreatment results, further confirmation in larger patient cohorts is necessary to validate our findings.

## Figures and Tables

**Table 1:** Clinicopathologic features of patients.

Parameters	Patients	Parameters	Patients

**Age**		**Node**	
**>**50	17	Positive	28
<50	16	Negative	5

**Menopause**		**ER**	
Pre-	17	ER (+)	23
Post-	16	ER (−)	10

**Grade**			
G0	22	**PR**	
G1	2	PR (+)	13
G2	6	PR (−)	20
G3	3		

**Molecular subtype**			
Luminal A	7	**HER2**	
Luminal B	16	HER2 (+)	12
TNBC	5	HER2 (−)	21
HER2 enriched	5		

**Histologic subtype**			
Invasive ductal carcinoma	26	**Lymphatic invasion**	
İnvasive lobular carcinoma	2	Positive	
Invasive breast carcinoma	2	Negative	13
Invasive mucinous carcinoma	1		6
Medullary carcinoma	2		

**Tumor size**			
T1	4	**Masculine invasion**	
T2	19	Positive	
T3	5	Negative	1
T4	4		18

**Table 2:** Pre- and post-NACT ccfDNA, mtDNAcn, CTC, EMT, ALDH1, and pathologic and clinical response.

			ccfDNA	mtDNA cn	CTC		EMT		ALDH1		Therapy response		Metastasis during
Patient no.	Molecular subgroup	Neoadjuvant chemotherapy regime	Pre-NACT	Post-NACT	Pre-NACT	Post-NACT	Pre-NACT	Post-NACT	Pre-NACT	Post-NACT	Pathologic	Clinical	
**P1**	Lum A	4′ EC + 12′ Paclitaxel	-	-	N		N		N		PR	CR	N
**P2**	Lum B	-	-	-	P	N	N	P	P	N	PR	PR	N
**P3**	Lum B, HER2^+^	-	-	-	N	P	N	N	N	N	PR	CR	N
**P4**	Lum B, HER2^+^	4′ EC + 12′ Paclitaxel + Herceptin	↑	↑	N	N	N	N	N	N	PR	PR	N
**P5**	Lum B, HER2^+^	12′ Paclitaxel + 4′ Herceptin + 4′ EC	↑	↑	N	N	N	N	N	N	CR	PR	N
**P6**	Lum B, HER2^+^	-	-	-	N		N		P		CR	PR	N
**P7**	Lum B	4′ EC + 12′ Paclitaxel	↑	↓	N	N	N	P	P	N	CR	CR	N
**P8**	Lum B	4′ EC + 12′ Paclitaxel	↑	↓	N	N	N	N	N	N	PR	PR	N
**P9**	Lum B	4′ EC + 12′ Paclitaxell	↑	↑	N	N	N	N	N	N	CR	CR	N
**P10**	Lum A	4′ EC + 12′ Paclitaxel	↓	↓	N	N	N	N	P	N	PR	PR	N
**P11**	TN	4′ EC + 9′ Paclitaxel	↓	↓	N	N	N	P	N	N	PR	PR	N
**P12**	Lum A	-	↑	↓	P	N	P	P	P	N	PR	PD	P
**P13**	Lum A	Anastrozole	↑	↑	N	N	N	N	N	N	PR	PR	N
**P14**	HER2 enriched		-	-	N	N	P	N	N	N	PR	PD	N
**P15**	Lum B, HER2^+^	4′ EC + 12′ Paclitaxel + 4′ Herceptin	↑	↓	N	N	N	N	N	N	PR	CR	N
**P16**	Lum B, HER2^+^	4′ EC + 12′ Paclitaxel + 4′ Herceptin	↑	↓	N	N	N	N	N	N	PR	PR	N
**P17**	Lum A	12′ Paclitaxel + 4′ EC	↑	↓	P	N	P	N	P	N	PR	PR	P
**P18**	Lum B	12′ Paclitaxel + 4′ AC	↑	↓	N	N	N	N	N	N	PR	PR	N
**P19**	TN	4′ AC + 10′ Paclitaxel	↓	↓	N	N	N	N	N	N	PR	PD	P
**P20**	Lum B, HER2^+^	-	-	-	N	N	N	N	N	P	PR	PR	N
**P21**	Lum A	4′ EC + 12′ Paclitaxel	↑	↓	N	N	N	N	N	N	PR	PR	N
**P22**	Lum B	4′ EC + 9′ Paclitaxel	↑	↓	N	N	N	N	N	N	CR	CR	N
**P23**	TN	4′ EC + 12′ Paclitaxel	↓	↑	N	N	N	N	N	N	CR	CR	N
**P24**	Lum B	4′ EC	↓	↓	N	N	N	P	N	P	PR	PR	N
**P25**	Lum B	4′ EC + 11′ Paclitaxel	↑	↓	N	N	N	N	N	N	PR	PR	N
**P26**	HER2 enriched	12′ Paclitaxel + 4′ Herceptin + 4′ AC	↑	↓	N	N	N	N	P	N	CR	CR	N
**P27**	Lum B	4′ AC	↑	↑	N	P	N	N	P	N	PR	PR	N
**P28**	TN	4′ EC + 12′ Paclitaxel	↑	↓	P	P	N	N	N	N	PR	PR	N
**P29**	HER2 enriched	12′ Paclitaxel + 6′ Herceptin + 4′ AC	-	-	N	N	N	N	N	P	CR	CR	P
**P30**	Lum B	4′ EC + 12′ Paclitaxel	↑	↓	P	N	N	N	P	N	PR	PR	P
**P31**	Lum B, HER2^+^	12′ Paclitaxel + 4′ Herceptin + 4′ AC	↑	↓	N	P	N	N	N	N	PR	PR	N
**P32**	Lum A	4′ AC + 9′ Paclitaxel	↓	↓	N	N	N	N	N	N	PR	PD	N
**P33**	HER2 enriched	10′ Paclitaxel + 7′ Herceptin + 4′ AC	↑	↑	N	P	N	N	N	N	CR	PR	N
**P34**	TN	4′ AC + 12′ Paclitaxel	↑	↑	N	P	N	N	N	N	PR	PR	P
**P35**	HER2 enriched	4′ AC + 12′ Paclitaxel + 4′ Herceptin	↑	-	N	N	N	N	N	N	CR	CR	N
**P36**	Lum B, HER2^+^	12′ Paclitaxel + 4′ Herceptin + 4′ AC	-	-	P		N		N		-	-	N

Abbreviations: ALDH1: tumor stem cells marker; CR: complete response; CTC: circulating tumor cell; EMT: epithelial-mesenchymal transition; HER2: Human epidermal growth factor receptor 2; Lum A: luminal A; Lum B; luminal B; NACT: neoadjuvant chemotherapy; N: negative; P: positive; PD: progressive response; PR: partial response; TN: triple negative.
